# Chromosomal changes in uroepithelial carcinomas

**DOI:** 10.1186/1475-9268-4-1

**Published:** 2005-08-07

**Authors:** Imad Fadl-Elmula

**Affiliations:** 1 Al Neelain Medical Research Center, Faculty of Medicine, Al Neelain University, Khartoum, Sudan

## Abstract

This article reviews and summarizes chromosomal changes responsible for the initiation and progression of uroepithelial carcinomas. Characterization of these alterations may lead to a better understanding of the genetic mechanisms and open the door for molecular markers that can be used for better diagnosis and prognosis of the disease. Such information might even help in designing new therapeutic strategies geared towards prevention of tumor recurrences and more aggressive approach in progression-prone cases.

The revision of 205 cases of uroepithelial carcinomas reported with abnormal karyotypes showed karyotypic profile characterized by nonrandom chromosomal aberrations varying from one or few changes in low-grade and early stage tumors to massively rearranged karyotypes in muscle invasive ones. In general, the karyotypic profile was dominated by losses of chromosomal material seen as loss of entire chromosome and/or deletions of genetic materials. Rearrangements of chromosome 9 resulting in loss of material from 9p, 9q, or of the entire chromosome were the most frequent cytogenetic alterations, seen in 45% of the cases. Whereas loss of material from chromosome arms 1p, 8p, and 11p, and gains of chromosome 7, and chromosome arm 1q, and 8q seem to be an early, but secondary, changes appearing in superficial and well differentiated tumors, the formation of an isochromosome for 5p and loss of material from 17p are associated with more aggressive tumor phenotypes. Upper urinary tract TCCs have identical karyotypic profile to that of bladder TCCs, indicating the same pathogenetic mechanisms are at work in both locales. Intratumor cytogenetic heterogeneity was not seen except in a few post-radiation uroepithelial carcinomas in which distinct karyotypic and clonal pattern were characterized by massive intratumor heterogeneity (cytogenetic polyclonality) with near-diploid clones and simple balanced and/or unbalanced translocations. In the vast majority of cases strong correlation between the tumors grade/stage and karyotypic complexity was seen, indicating that progressive accumulation of acquired genetic alterations is the driving force behind multistep bladder TCC carcinogenesis. Although most of these cytogenetic alterations have been identified for many years, the molecular consequences and relevant cancer genes of these alterations have not yet been identified. However, loss of TSG(s) from chromosome 9 seems to be the primary and important event(s) in uroepithelial carcinogenesis

## Introduction

Uroepithelial cancer e.g., cancer of the urinary bladder, ureter, and renal pelvis, is the 4^th ^and 8^th ^most common malignant neoplasm in men and the women respectively [1]. In areas where infestation with *Schistosoma haematobium *is endemic, uroepithelial cancer is even more common and accounts for 25% of all cancers in men. The incidence, the etiologies, and the histology types vary considerably among countries. In Europe and the USA, chemicals, including smoking, are the main etiology of dominant transitional cell carcinoma (TCC) (90%) of the bladder. The picture is reversed in the Middle East and Africa, where urinary *Schistosoma haematobium *is associated with an increased risk of developing BC, especially of the SCC type. In these areas BC is the most common cancer in men (25% of all cancers) [2].

Uroepithelial cancer is characterized by unique natural history that is characterized by heterogeneity of the clinical course seen as variation in clinical-course of tumors [3]. Even tumors of the same pathologic stage, may follow very different clinical courses. The prognostic markers currently used are tumor grade, multiplicity, tumor shape, location, and presence of carcinoma *in situ *(Cis); however all are of limited value [4,5]. The difficulties encountered in predicting the clinical course, especially for superficial bladder tumors may suggest that TCC has at least two main subgroups with distinct genetic make-ups and, consequently, clinical behavioral patterns [6]. The natural history is also characterized by high incidence of recurrence seen in 70% of the cases after the initial treatment even in superficial bladder tumors [3,7]. Ureteral and renal pelvis tumors also have a high risk (40%–75%) of having recurrent bladder tumor and 50% of these cases develop metastasis [8]. Of these, 80% remain superficial throughout the patient's life, whereas 16% to 25% eventually recur as higher grade and/or stage tumor(s) [9]. The last clinical characteristics of uroepithelial carcinomas is the multifocal nature of the disease, seen in around 30% of urinary tract TCC at the time of diagnosis [5]. Although the mechanism behind the polychronotopicity of uroepithelial carcinomas is settling, since the possibility of new tumor(s) arising as the result of field cancerization was ruled out by cytogenetic analysis of synchronous and/or metachronous tumors [10], the heterogeneity of the clinical course justify the need for the study of prognostic genetic marker. Such information when made available might help in the design of new therapeutic strategies geared toward a more aggressive multimodal treatment approach in progression-prone cases.

## Materials and methods

A total of 205 uroepithelial tumors (188 bladder tumors, 15 ureteral, one renal pelvis and one prostatic urethra tumor) were obtained from 153 male and 48 female patients. They were processed for short-term culturing and chromosomal analysis after staining with Wright stain according to the standard protocols. All cases revealed cytogenetic abnormality and all were reported previously [Appendix 1]. Of these only 3 tumors were SCC, whereas the remaining were of TCC type. The histopathology examination revealed superficial disease (Ta-1/GI-II) in 109 tumors and muscle invasion (T2-or more) in 96 tumors. For each case the histology grading, the tumor staging, and the karyotypic descriptions were reviewed in order to understand chromosomal changes and cytogenetic profile associated with biological parameters of uroepithelial tumors.

### Maps of breakpoints and imbalances

**Figure 1:**
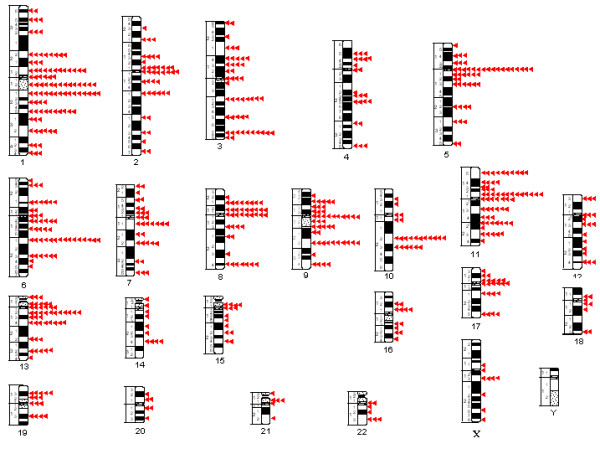
Distribution of 677 breakpoints observed in structural chromosomal aberrations in 205 uroepithelial carcinomas.

**Figure 2:**
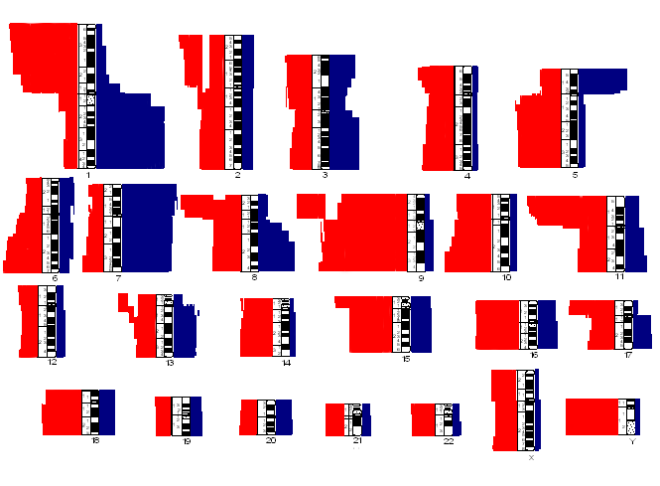
Karyotypic imbalances caused by numerical and structural aberrations in 205 uroepithelial carcinomas. Losses are shown in red, gains in blue.

To help identify chromosomal changes in a more comprehensive manner, the data were also presented as breakpoint and karyotypic imbalance maps (Figures 1 and 2). In order to report the basic and most representative range of cytogenetic changes and to avoid cytogenetic noise brought about by possible ploidy shifts, we included only 2n-4n clones for each tumor in the maps. In case of duplicated aberrations found within a clone or in more than one related clone, the breakpoints were plotted once only. If any aberrant chromosome was involved twice in the same clone or in a related clone, only additional breakpoints were considered. When the same chromosome was involved in both numerical and structural aberrations, we recorded only the total net imbalance, and if the same imbalance was found in more than one related clone, it was recorded only once. If additional gains or losses of the same chromosome or chromosomal segment were found in related clones, only the largest imbalance was recorded.

## Results

A total of 205 uroepithelial carcinomas have been reported with chromosomal changes.(Appendix 1). Of these, 40 (18%) had grossly incomplete karyotypes, 58 (26%) displayed altogether 157 unidentified marker chromosomes as well as 18 unidentified ring chromosomes and a total of 208 uncertain breakpoint, as is evident from how the karyotypes were written [11].

All chromosomes were involved in numerical and/or structural aberrations at least once. The most commonly involved were chromosomes 9 (in 45%) of cases, 1 (in 29%), 11 (in 27%), 8 (in 22%), 7 (in 20%), Y (in 20%), 5 (in 18%), 17 (in 18%), 3 (in 17%), 15 (in 15%), 6 (in 13%), 14 (in 13%), and 18 (in 12%).

A total of 15 unbalanced recurrent chromosomal rearrangements were described: del(1)(q21), i(1)(q10), i(5)(p10), del(6)(q21), i(11)(q10), del(1)(p11), add(3)(q21), add(5)(q11), del(6)(q13), i(13)(q10), del(14)(q24), and i(17)(q10).

A total of 677 chromosomal breakpoints were identified in the 205 uroepithelial TCC showing structural chromosomal abnormalities (Figure 1), with the highest number of breakpoints (95) seen in chromosome 1 and more than 40 seen in chromosomes 2, 3, 5, 6, 8, 9, 11, and 13. The chromosome bands most frequently involved were 1p12, 1q12, 2q21, 1q25, 2q27, 5p10, 6q21, 9p10, 9q22, 10q22, 11p15, 11p11 and 13q12 (each involved at least 10 times). The karyotypic imbalances resulting from the structural and numerical chromosomal aberrations (Figure 2) were dominated by loss of an entire copy of chromosomes 4, 9, 10, 14, 15, 16, 18, 21, 22, X, and Y, and gain of entire copy of chromosomes 7, 16, 19, and 20. Losses of the entire arms or parts of 1p, 5q, 8p, 9p, 11p, 13p, 15p and 17p and gain of 1q, 3q, 5p, 8q, 13q, and 17q were also common.

## Discussion

Although uroepithelial carcinomas are among the most common malignancies, their karyotypic characteristics and genetic pathway remain poorly understood. Most of the reported data are quantitatively limited and lacking karyotypic precision [11]

Although no specific chromosomal aberration has been identified for uroepithelial carcinomas, a clearly nonrandom pattern of chromosomal changes has emerged, albeit with considerable karyotypic heterogeneity among cases ranging from the presence of sole anomalies in early tumors to very complex karyotypes in advanced ones. Translocations are rarely seen, at least in early stages, and seem to play no important role in the initiation of uroepithelial carcinomas. Instead, the cytogenetic profile is dominated by nonrandom chromosome gains and, especially, losses, the latter indicating that loss of tumor suppressor gene(s) may be the most crucial event in the pathogenesis of uroepithelial carcinomas.

The fact that chromatin losses dominated the imbalance pattern indicates that loss of tumor suppressor gene(s) is the most important pathogenetic consequence of the chromosomal aberrations associated with uroepithelial carcinoma.

**Figure 3:**
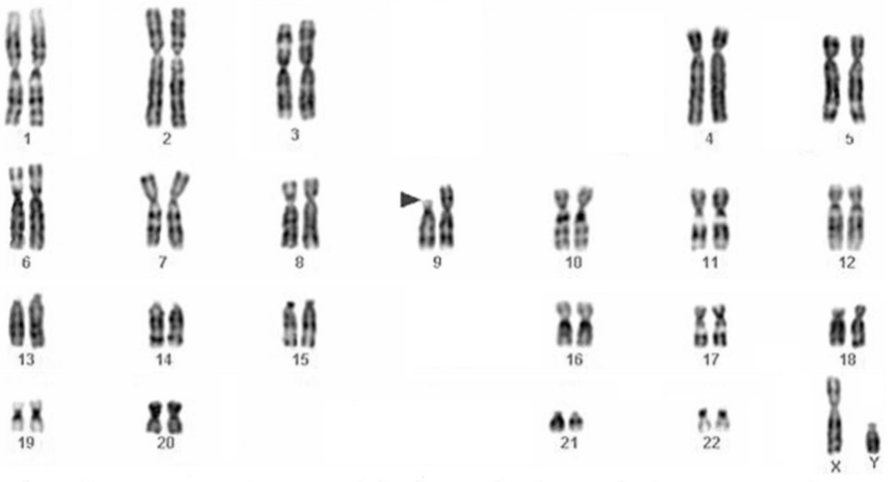
Representative karyotype from a well differentiated transitional cell carcinoma of the bladder. The arrowhead indicates the breakpoint in a deleted chromosome 9 {46,XY,del(9)(p11)}.

**Figure 4:**
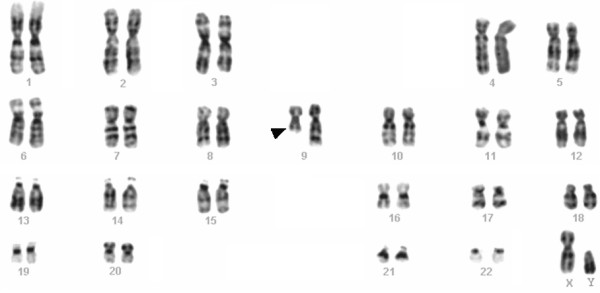
Representative karyotype from a well differentiated transitional cell carcinoma of the bladder. The arrowhead indicates the breakpoint in a deleted chromosome 9 {46,XY,del(9)(q12q22)}.

Changes involving chromosome 9 [-9, del(9p), del(9q)] are the most common chromosomal aberrations in uroepithelial carcinomas. Rearrangements of chromosome 9 are seen as the sole change in cases with simple karyotypes in early and superficial carcinomas but also persisted in the massively complex karyotypes of advanced muscle invasive tumors [12]. Besides loss of the entire chromosome copy, losses of material from either arms is often seen, which may indicates the presence of at least one pathogenetically important TSG in each arm [13,14] (Figures 3 and 4). Loss of chromosome 9 material had therefore been widely accepted as an early ubiquitous, pathogenetically important and early event in urinary tract transitional cell carcinogenesis [15,16]. Recent data suggest that 9q abnormalities are more common in Ta compared with T1 tumors, in which a mixture of aberrant 9p and 9q genotypes are seen [17]. These observations indicate that loss of 9p material may be associated with the development of tumors with more aggressive biological behavior or, alternatively, they may be related to early disease progression [6]. Although several attractive candidates such as p16/CDKN2 in 9p21 and TSC1 in 9q34 have been reported to be homozygously deleted in superficial TCC of the bladder [18] the crucial gene-level consequences of these chromosomal aberrations remain unknown.

**Figure 5:**
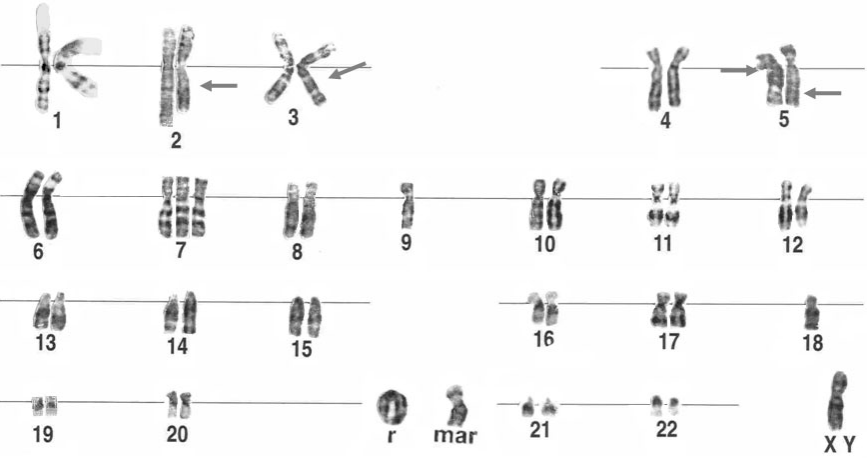
Representative karyotype from a well differentiated transitional cell carcinoma of the bladder. The chromosome indicated by "mar" represents unidentified marker, "r" represents ring chromosome. Arrowheads indicate breakpoints.

Trisomy 7 has been frequently described in near-diploid karyotypes and as the sole chromosomal change in bladder and ureteral tumors [19] (Figure 5). The importance of trisomy 7 as a tumor-associated aberration and its molecular consequence remain controversial since it has been found repeatedly also in some unquestionably nonneoplastic lesions [20]. However, the important molecular consequences of trisomy 7 may be an increased number of alleles for the epidermal growth factor (EGF) receptor gene.

In addition to chromosomes 9 and 7, chromosomes 1, 8, and 11 seemed to be preferentially involved and were found to be rearranged in superficial tumors. Alterations involving chromosome 1 had been seen in 35% of the investigated BC cases [11]. The alterations are diverse and include deletions, translocations, duplications, and isochromosome formations. However, regardless of the type of changes, the most common consequences of the changes in chromosome 1 were gain of 1q material and loss of 1p material. Changes involving chromosome 1 are rarely if ever seen as the sole aberration but nevertheless may form part of relatively simple karyotypes. Support for the view that changes of chromosome 1 are secondary in BC tumorigenesis also comes from molecular cytogenetic investigations showing that gain of 1q material is more frequent in pT1 than in pTa tumors [21]. Regardless of when they occur, the molecular consequences and biological significance of these changes in uroepithelial carcinomas remain uncertain, although some investigators suggest that they may be implicated in tumor progression and in vitro immortalization of human cells [22].

Aberrations involving chromosome 8 usually resulting in loss of material from the short arm, have been repeatedly detected in urinary tract tumors. Losses from 8p and gain of 8q were the dominant net result in, almost all, changes affecting this chromosome. Although often found in simple karyotypes of low-grade tumors, the aberrations involving chromosomes 1, 8 and/or 11 were never the sole change, but typically accompanied by chromosome 9 rearrangements [11]. Similar aberrations occur frequently also in many other tumor types as carcinoma of the lung[23]. Studies of patients with non-muscle-invasive bladder cancers have shown more frequent loss of 8p in minimally invasive (pT1) tumors than in non-invasive (pTa) ones [24]. These findings are consistent with the view that loss of a putative TSG on 8p plays important role in the progression and tumor invasion in BC.

Several cytogenetic studies have revealed nonrandom involvement of chromosome 11 in BC, mostly leading to loss of genetic material from 11p [11]. Additional evidence to the same effect has come from CGH analyses[25]. The frequency of 11p loss may be higher in pT1 than in pTa tumors, and even higher in pT2-4b tumors [26]. In contrast, some FISH studies using centromeric probes have shown an increased copy number of chromosome 11[27]. However, these studies did not assess the tumors' ploidy level and the seemingly increased copy number may therefore have reflected general polyploidization. Loss of 11p therefore appears to be an early but secondary change, associated with tumor progression. The putative TSG on 11p lost through the chromosomal rearrangements remain unknown.

### Chromosomal changes associated with aggressive biological tumor behavior

The formation of an isochromosome for 5p, i(5)(p10), leading to net gain of material from the short arm of chromosome 5 has been reported in bladder TCCs, making it the single most common recurrent structural chromosomal abnormality in Uroepithelial tumors [11]. In most cytogenetic reports the aberration was associated with aggressive tumor phenotypes, muscle-invasive and poorly differentiated tumors. Considering the fact that this marker has not been consistently linked to any other malignancy and that it is seldom seen in association with rearrangements of chromosome 17, it appears to characterize a subgroup of advanced TCC of the bladder arising via a unique pathogenetic pathway. A strong correlation between chromosome 5 involvement and tumor grade and stage was shown in several studies [6,28].

Rearrangement of chromosome 17 resulting in loss of material from the short arm is rarely seen in superficial BC, but is common in more advanced and aggressive tumors. The molecular target of 17p material loss could be the *TP53 *gene, which resides at 17p12, and is known as the most generally important tumor suppressor gene in human neoplasia. Cytogenetic analyses revealed involvement of 17p in less than 10% of the analyzed tumors, and molecular genetic studies have shown LOH in up to 42% of the tumors. While cytogenetic data probably underestimate 17p involvement. However, this finding indicates that most deletions of 17p are submicroscopic and thus cannot be detected using conventional cytogenetic technique. On the other hand, several FISH analyses using centromeric probes have revealed frequent chromosome 17 copy-gain in bladder carcinomas of high grade and stage. Such apparent gains of whole chromosome copies in FISH analyses may reflect polyploidization in advanced tumors rather than a specific role of chromosome 17. Although rearrangement of chromosome 17 resulting in loss of material from the short arm was seen frequently in high-grade, muscle-invasive tumors, loss of 17p material is not restricted only to advanced TCC [12]. The loss of 17p material in some superficial tumors fits the prognostic heterogeneity seen even in superficial BC in which some tumors take a more aggressive clinical course. For these ones it has been suggested that early loss of 17p, combined with mutational inactivation of the *TP53 *allele on the other chromosome 17, could be responsible for the aggressive behavior seen in 16–25% of superficial BCs.

Rearrangement of chromosome 3 resulting in loss of material from the short arm had been seen in 44% of the reported BC with abnormal karyotypes, usually in advanced tumors with complex karyotypes [29]. Deletion of 3p is also frequent in other carcinomas such as renal cell cancer, breast cancer, and small cell lung cancer [11]. Although many potential target genes are located on 3p, the crucial molecular-level consequence of these chromosomal aberrations in BC carcinogenesis remains unknown.

Aberrations involving chromosome 13 are known to occur in muscle invasive tumors and often result in net loss of material from the long arm [11]. LOH studies seem to be more informative than cytogenetic studies in this prospect as LOH at the RB locus in band 13q14 was seen in 90% of muscle invasive tumors.

Loss of the Y chromosome is common in bladder tumors of male patients. It has been reported in all stages and even as the sole change in several reports [30]. In contrast, bladder tumors obtained from female patients do not reveal the same incidence of X chromosome loss. FISH studies have shown that loss of the Y is infrequent in normal urothelial cells obtained from healthy males [31]. This observation does not exclude, however, the possibility that loss of chromosome Y in cultured cells could reflect changes in stromal elements, in particular since-Y has been demonstrated in non-neoplastic disease lesions of several tissues and organs such as kidney, bone marrow, and brain [32,33]. In conclusion, one cannot be certain that loss of the Y chromosome really signifies a pathogenetically important event in neoplastic cells.

### Correlation between karyotype and tumor grade and stage

Strong correlation was seen between the grade/stage of the tumors and the karyotypic profile. Most superficial and well-differentiated tumors (TaG1) were pseudo-or near-diploid and exhibited simple karyotypes (5 or fewer chromosomal changes) (Figures 3, 4, 5). A progressive increase in the number of chromosome aberrations with tumor grade and/or stage was evident in most large reported series, with TaG1 tumors showing less abnormal karyotypes than did those that were T1G2, which, in their turn, were less abnormal than T2G3 tumors. This is in agreement with the view that the uroepithelial carcinomas follow the multistep carcinogenesis and that their clinical progression is steered by the synergistic effect of accumulated genetic alterations.

### Upper urinary tract carcinomas (UUTC)

Very limited information is available on cytogenetics of UUTC; only 16 cases with abnormal karyotype, have been published [34,35]. Because of the great similarities between upper urinary tract carcinomas (UUTC) and bladder TCC with regard to etiology, histology, and natural disease history, one would assume that the tumorigenic mechanisms, including the karyotypic profile, are more or less identical.

Loss of material from chromosome 9 was seen in most informative UUTC [12]. The ubiquity of chromosome 9 involvement thus seems to be no less pronounced in UUTC than BC; this is in agreement with the view that this is an early and crucial event in the genesis of nearly all urinary tract TCC, regardless of their site and stage. The high frequency of chromosome 9 involvement in upper urinary tract transitional cell carcinogenesis makes this a logical target for a possible genetic marker to be used in the follow-up of patients with such tumors, something that would be particularly useful considering how frequent downstream disease spreading is in these patients.

### Post-radiation uroepithelial carcinomas

Post-radiation tumors or second-primary radiation-induced cancers have distinct clinical and cytogenetic characteristics. These tumors develop in an irradiated field and should have a different histological type from that of the primary tumors. Usually they are infiltrative and of high grade at the time of diagnosis. Only 2 cases of post-radiation uroepithelial carcinoma with cytogenetics abnormality have been reported [35,36]. The karyotypic profile in both cases revealed near-diploid, karyotypically abnormal clones characterized by rather simple balanced and/or unbalanced translocations in each tumor. The intratumor karyotypic heterogeneity seen in these cases indicates massive polyclonality in contrast to the cytogenetic monoclonality consistently demonstrated in urinary tract TCC. This reflects the likelihood of the previous radiation carcinogenic effect and illustrating that post-radiation urinary tract tumors are distinct from other urinary tract TCC not only etiologically but also with regard to the pathogenetic mechanisms involved.

## Supplementary Material

Additional File 1 Clinical and cytogenetic data on 205 cases of uroepithelial carcinomas Click here for file

## References

[B1] Silverman DT, Hartge P, Morrison AS, Devesa SS (1992). Epidemiology of bladder cancer. Hematol Oncol Clin North Am.

[B2] Badawi AF, Mostafa MH, Probert A, O'Connor PJ (1995). Role of schistosomiasis in human bladder cancer: evidence of association, aetiological factors, and basic mechanisms of carcinogenesis. Eur J Cancer Prev.

[B3] Herr HW (1997). Natural history of superficial bladder tumors: 10- to 20-year follow-up of treated patients. World J Urol.

[B4] Aprikian AG, Sarkis AS, Reuter VE, Cordon-Cardo C, Sheinfeld J (1993). Biological markers of prognosis in transitional cell carcinoma of the bladder: current concepts. Semin Urol.

[B5] Holmang S, Hedelin H, Anderstrom C, Johansson SL (1995). The relationship among multiple recurrences, progression and prognosis of patients with stages Ta and T1 transitional cell cancer of the bladder followed for at least 20 years. J Urol.

[B6] Hoglund M, Sall T, Heim S, Mitelman F, Mandahl N, Fadl-Elmula I (2001). Identification of cytogenetic subgroups and karyotypic pathways in transitional cell carcinoma. Cancer Res.

[B7] Lee R, Droller MJ (2000). The natural history of bladder cancer. Implications for therapy. Urol Clin North Am.

[B8] Steffens J, Nagel R (1988). Tumours of the renal pelvis and ureter. Observations in 170 patients. Br J Urol.

[B9] Kiemeney LA, Witjes JA, Heijbroek RP, Verbeek AL, Debruyne FM (1993). Predictability of recurrent and progressive disease in individual patients with primary superficial bladder cancer. J Urol.

[B10] Fadl-Elmula I, Gorunova L, Mandahl N, Elfving P, Lundgren R, Mitelman F, Heim S (1999). Cytogenetic monoclonality in multifocal uroepithelial carcinomas: evidence of intraluminal tumour seeding. Br J Cancer.

[B11] Mitelman F (2008). www.Mitelmansdatabase.org.

[B12] Fadl-Elmula I, Gorunova L, Mandahl N, Elfving P, Lundgren R, Mitelman F, Heim S (2000). Karyotypic characterization of urinary bladder transitional cell carcinomas. Genes Chromosomes Cancer.

[B13] Ohgaki K, Minobe K, Kurose K, Iida A, Habuchi T, Ogawa O, Kubota Y, Akimoto M, Emi M (1999). Two target regions of allelic loss on chromosome 9 in urinary-bladder cancer. Jpn J Cancer Res.

[B14] Simoneau AR, Spruck CH, Gonzalez-Zulueta M, Gonzalgo ML, Chan MF, Tsai YC, Dean M, Steven K, Horn T, Jones PA (1996). Evidence for two tumor suppressor loci associated with proximal chromosome 9p to q and distal chromosome 9q in bladder cancer and the initial screening for GAS1 and PTC mutations. Cancer Res.

[B15] Cairns P, Shaw ME, Knowles MA (1993). Initiation of bladder cancer may involve deletion of a tumour-suppressor gene on chromosome 9. Oncogene.

[B16] Simoneau M, LaRue H, Aboulkassim TO, Meyer F, Moore L, Fradet Y (2000). Chromosome 9 deletions and recurrence of superficial bladder cancer: identification of four regions of prognostic interest. Oncogene.

[B17] Gonzalez-Zulueta M, Shibata A, Ohneseit PF, Spruck CH, Busch C, Shamaa M, El-Baz M, Nichols PW, Gonzalgo ML, Elbaz M, al. (1995). High frequency of chromosome 9p allelic loss and CDKN2 tumor suppressor gene alterations in squamous cell carcinoma of the bladder [published erratum appears in J Natl Cancer Inst 1995 Dec 6;87(23):1807]. J Natl Cancer Inst.

[B18] Nishiyama H, Takahashi T, Kakehi Y, Habuchi T, Knowles MA (1999). Homozygous deletion at the 9q32-33 candidate tumor suppressor locus in primary human bladder cancer. Genes Chromosomes Cancer.

[B19] Berrozpe G, Miro R, Caballin MR, Salvador J, Egozcue J (1990). Trisomy 7 may be a primary change in noninvasive transitional cell carcinoma of the bladder. Cancer Genet Cytogenet.

[B20] Johansson B, Heim S, Mandahl N, Mertens F, Mitelman F (1993). Trisomy 7 in nonneoplastic cells. Genes Chromosomes Cancer.

[B21] Richter J, Wagner U, Schraml P, Maurer R, Alund G, Knonagel H, Moch H, Mihatsch MJ, Gasser TC, Sauter G (1999). Chromosomal imbalances are associated with a high risk of progression in early invasive (pT1) urinary bladder cancer. Cancer Res.

[B22] Terracciano L, Richter J, Tornillo L, Beffa L, Diener PA, Maurer R, Gasser TC, Moch H, Mihatsch MJ, Sauter G (1999). Chromosomal imbalances in small cell carcinomas of the urinary bladder. J Pathol.

[B23] Wistuba II, Behrens C, Virmani AK, Milchgrub S, Syed S, Lam S, Mackay B, Minna JD, Gazdar AF (1999). Allelic losses at chromosome 8p21-23 are early and frequent events in the pathogenesis of lung cancer. Cancer Res.

[B24] Zhao J, Richter J, Wagner U, Roth B, Schraml P, Zellweger T, Ackermann D, Schmid U, Moch H, Mihatsch MJ, Gasser TC, Sauter G (1999). Chromosomal imbalances in noninvasive papillary bladder neoplasms (pTa). Cancer Res.

[B25] Voorter C, Joos S, Bringuier PP, Vallinga M, Poddighe P, Schalken J, du Manoir S, Ramaekers F, Lichter P, Hopman A (1995). Detection of chromosomal imbalances in transitional cell carcinoma of the bladder by comparative genomic hybridization. Am J Pathol.

[B26] Sauter G, Gasser TC, Moch H, Richter J, Jiang F, Albrecht R, Novotny H, Wagner U, Bubendorf L, Mihatsch MJ (1997). DNA aberrations in urinary bladder cancer detected by flow cytometry and FISH. Urol Res.

[B27] Hopman AH, Moesker O, Smeets AW, Pauwels RP, Vooijs GP, Ramaekers FC (1991). Numerical chromosome 1, 7, 9, and 11 aberrations in bladder cancer detected by in situ hybridization. Cancer Res.

[B28] von Knobloch R, Bugert P, Jauch A, Kalble T, Kovacs G (2000). Allelic changes at multiple regions of chromosome 5 are associated with progression of urinary bladder cancer. J Pathol.

[B29] Knowles MA, Elder PA, Williamson M, Cairns JP, Shaw ME, Law MG (1994). Allelotype of human bladder cancer. Cancer Res.

[B30] Smeets W, Pauwels R, Laarakkers L, Debruyne F, Geraedts J (1987). Chromosomal analysis of bladder cancer. III. Nonrandom alterations. Cancer Genet Cytogenet.

[B31] Bentz M, Plesch A, Stilgenbauer S, Dohner H, Lichter P (1998). Minimal sizes of deletions detected by comparative genomic hybridization. Genes Chromosomes Cancer.

[B32] Elfving P, Cigudosa JC, Lundgren R, Limon J, Mandahl N, Kristoffersson U, Heim S, Mitelman F (1990). Trisomy 7, trisomy 10, and loss of the Y chromosome in short-term cultures of normal kidney tissue. Cytogenet Cell Genet.

[B33] Heim S, Mandahl N, Jin Y, Stromblad S, Lindstrom E, Salford LG, Mitelman F (1989). Trisomy 7 and sex chromosome loss in human brain tissue. Cytogenet Cell Genet.

[B34] Batata M, Grabstald H (1976). Upper urinary tract urothelial tumors. Urol Clin North Am.

[B35] Fadl-Elmula I, Gorunova L, Mandahl N, Elfving P, Lundgren R, Rademark C, Heim S (1999). Cytogenetic analysis of upper urinary tract transitional cell carcinomas. Cancer Genet Cytogenet.

[B36] Fadl-Elmula I, Bonaldi L, Gorunova L, Mandahl N, Elfving P, Heim S (1998). Cytogenetic heterogeneity in a second primary radiation-induced bladder carcinoma: ten karyotypically unrelated clones. Cancer Genet Cytogenet.

